# Effect of Quenching and Tempering and Austempering Treatments on the Microstructural Evolution and Mechanical Properties of High-Strength Bolt Steel

**DOI:** 10.3390/ma19204314

**Published:** 2026-10-13

**Authors:** Ziwen Li, Zhenguo Hou, Jie Yan, Min Yang, Luliang Zhao, Chunqiao Xing, Zan Yao

**Affiliations:** 1 School of Materials Science and Engineering, Shanghai University, Shanghai 200444, China18115694113@163.com (L.Z.); 18642515957@163.com (C.X.); 2 Composite Research Center, Shanghai University, Shanghai 200444, China; ym2008@shu.edu.cn; 3 State Key Laboratory of Advanced Refractories, Shanghai University, Shanghai 200444, China

**Keywords:** quenching and tempering, austempering, bainite, tempered sorbite, mechanical properties

## Abstract

This study systematically compares the effects of quenching and tempering (Q&T) and austempering on the microstructural evolution and mechanical properties of 42CrMo and 40CrNiMo high-strength bolt steels. The steels were subjected to Q&T or austempering after austenitization, and their microstructures, tensile properties, hardness, and impact behavior were comparatively evaluated. Q&T produced tempered martensite/tempered sorbite with fine and uniformly distributed carbides, whereas austempering produced a bainitic lath structure characterized by fewer and coarser carbides, larger effective grain sizes, and heterogeneous carbon distribution. At comparable strength levels, austempering resulted in lower yield strength and improved tensile ductility compared with Q&T, while substantially reducing the absorbed impact energy of both steels. Microstructural and fractographic analyses indicate that the different mechanical responses are closely associated with carbide precipitation and carbon distribution, while easier crack initiation and propagation along bainite blocks or lath interfaces contribute to the inferior impact performance of the austempered specimens. These findings clarify the microstructure-property relationships of Q&T and austempered high-strength bolt steels and provide a basis for optimizing heat-treatment strategies to achieve an appropriate balance of strength, ductility, and impact performance.

## 1. Introduction

High-strength low-alloy steels are widely used in critical components for engineering machinery, rail transit, and bridge construction, owing primarily to their superior mechanical properties and attractive cost-effectiveness. Bolted joints are among the most widely used mechanical joining methods in industrial applications, and high-strength bolts are commonly manufactured from CrB-, CrMo-, and CrNiMo-based low-alloy steels. With the continued development of modern equipment toward light weight, higher load-bearing capacity, and extended service life, structural materials are required to provide not only high strength but also adequate ductility and impact resistance. However, there is often a trade-off between material strength and ductility. Although conventional strengthening strategies can effectively increase resistance to plastic deformation, they may also reduce resistance to crack initiation and propagation [1,2,3]. Therefore, achieving a favorable balance among strength, ductility, and toughness while maintaining a high strength level has become an important objective in the microstructural design of low-alloy steels.

Among high-strength bolt steels, 42CrMo and 40CrNiMo are representative Cr-based alloy structural steels [4,5]. As a typical Cr-Mo alloy structural steel, 42CrMo is widely used for manufacturing grade 10.9 and 12.9 high-strength bolts because of its high strength and good hardenability. Compared with Cr-Mo steels, the addition of Ni to 40CrNiMo improves hardenability and enhances the ductility and toughness. However, due to its higher alloying costs, it is typically used to manufacture critical fasteners for more complex operating environments and larger dimensions.

At present, Q&T is the dominant industrial heat treatment process for manufacturing high-strength bolts. By quenching to obtain supersaturated martensite, followed by carbon diffusion, carbide precipitation, and dislocation recovery during the tempering process, a tempered sorbite microstructure with good comprehensive properties can be formed [6,7,8]. In this structure, uniformly dispersed carbides can impede dislocation motion and increase crack propagation resistance, enabling quenched and tempered steel to achieve excellent strength-toughness matching. However, increasing the tempering temperature promotes carbide coarsening, which reduces the strength and may adversely affect the ductility and toughness. In recent years, austempering has attracted extensive attention because it can regulate the bainite transformation process and obtain a refined bainite microstructure. Previous studies have shown that controlling the isothermal transformation temperature and holding time can refine bainitic ferrite laths and regulate the stability of retained austenite, thereby obtaining a multiphase microstructure with bainite as the main constituent, containing martensite and retained austenite, which ultimately improves the strength and toughness [9,10,11,12]. However, previous studies on austempering have mainly focused on ultrafine bainitic microstructures produced under low-temperature and long-duration conditions [13,14,15]. The austempering behavior of medium-carbon high-strength bolt steels at intermediate temperatures, particularly the relationship between microstructure and the resulting strength and toughness, remains insufficiently understood. Although bainitic microstructures are generally considered beneficial for improving the strength–toughness combination of steels, their mechanical properties depend strongly on the transformation temperature, bainite morphology, carbon redistribution, and precipitation behavior. In particular, a refined bainitic structure does not necessarily lead to superior impact toughness when microstructural heterogeneity or coarse precipitates is present.

Previous work has established the strong dependence of bainitic properties on the transformation temperature, morphology, carbon redistribution, retained-austenite stability, and precipitation behavior [9,10,11,12,13,14,15]. Nevertheless, most studies emphasize low-temperature nanobainite, individual steel grades, or comparisons made at different strength/hardness levels. Consequently, the practical question of whether intermediate-temperature austempering can replace conventional Q&T in medium-carbon high-strength bolt steels at a comparable hardness remains insufficiently resolved. This gap is particularly relevant, because hardness alone is often used as a rapid engineering indicator, although it does not uniquely determine yield behavior, tensile ductility, or resistance to dynamic crack propagation.

To address these issues, this study selects 42CrMo and 40CrNiMo steel as research objects and adopts Q&T and austempering as two heat treatment processes to systematically investigate their effects on the microstructure evolution and mechanical properties of the tested materials. Mechanical testing, including tensile, impact, and hardness tests, together with SEM, EBSD, and TEM characterization, was used to elucidate the differences between bainitic and tempered sorbite microstructures in terms of the grain structure, carbide precipitation, elemental distribution, and crack propagation behavior. Particular attention is given to the microstructural mechanisms underlying the changes in ductility and toughness of austempered specimens at strength levels comparable to those of Q&T specimens. This research provides a theoretical basis for the strengthening and toughening design and heat treatment process optimization of high-strength low-alloy structural steels. By directly comparing two steels and two heat-treatment routes at comparable hardness, this study reveals a consistent divergence between the tensile and impact responses across both steels and associates it with differences in carbide precipitation and crack propagation.

## 2. Materials and Methods

### 2.1. Materials

The chemical compositions of the 42CrMo and 40CrNiMo steels used in this study are listed in Table 1.

**Figure 1:**
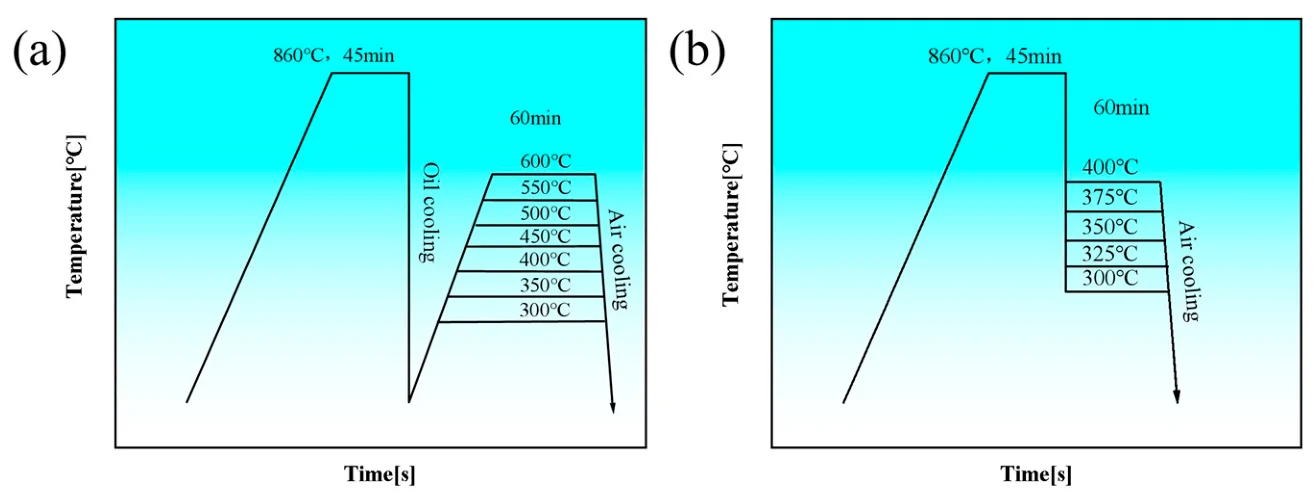
Schematic diagrams of the (**a**) quenching and tempering and (**b**) austempering (AT) processes.

The 42CrMo and 40CrNiMo steels used in this study were supplied as round bars with a diameter of 27 mm. The initial state is hot-rolled, and the microstructure consists of ferrite and pearlite. Both steels were austenitized in a muffle furnace at 860 °C for 45 min at a heating rate of 10 °C/s, followed by either oil quenching and tempering or austempering. The tempering temperature for the Q&T heat treatment ranged from 300 °C to 600 °C (in 50 °C increments), with a holding time of 60 min. Austempering was performed in a salt bath furnace, with an isothermal temperature range of 300 °C to 400 °C (in 25 °C increments) and a holding time of 60 min. The specimens for Q&T heat treatment of 42CrMo and 40CrNiMo steels were designated as QT-42 and QT-40, respectively, while those for austempering were designated as AT-42 and AT-40. The specific heat treatment processes are shown in Figure 1. To examine whether the low absorbed impact energy of the austempered condition could be recovered by a subsequent tempering step, selected specimens austempered at 375 °C for 60 min were additionally tempered at 450 °C for 60 min. These conditions are designated ATT-42 and ATT-40 for 42CrMo and 40CrNiMo, respectively.

### 2.2. Mechanical Tests

The hardness of samples subjected to different heat treatments was measured using a micro-Vickers hardness tester (HV-1000MPTA, Shanghai Hengyi Precision Instrument Co., Ltd., Shanghai, China) with a load of 1 kg and a dwell time of 5 s. For each heat-treatment condition, five measurements were performed at different locations, and the average value was reported. The tempering and austempering temperatures corresponding to comparable hardness levels were then selected for subsequent microstructural and mechanical characterization. Charpy impact specimens were machined from an off-center position of the original round bars, with their longitudinal axes parallel to the bar axis. Charpy impact specimens with dimensions of 10 mm × 5 mm × 55 mm and a V-notch were machined, and room-temperature impact tests were performed using a pendulum impact testing machine (JB-300B, ZwickRoell GmbH & Co. KG, Ulm, Germany) with a nominal energy capacity of 450 J. The tests were conducted in accordance with GB/T 229-2020 [16]. The sub-size specimens contained a 45° V-notch with a notch depth of 2 mm and a root radius of 0.25 mm. Two independent specimens were tested for each heat-treatment condition, and the measured results are reported as absorbed impact energy (J). The 10 mm × 5 mm × 55 mm specimens used in this study were half-thickness Charpy specimens. Therefore, the absorbed impact energy values reported herein are intended for comparative evaluation among specimens with identical geometry and dimensions and should not be directly compared with those obtained from full-size 10 mm × 10 mm × 55 mm Charpy specimens. Room-temperature tensile tests were performed on a WDW-200 universal testing machine (Lishi (Shanghai) Instrument Co., Ltd., Shanghai, China) in accordance with GB/T 228.1-2021 [17]. The round specimens had a parallel-section diameter of 8 mm and a gauge length of 70 mm. Two specimens were tested for each heat-treatment condition. For Method A2, the standard-recommended estimated strain rates are 2.5 × 10^−4^ s^−1^ for determining Rp0.2 and 6.7 × 10^−3^ s^−1^ for determining Rm and ductility properties. The standard specifies class 1 or better accuracy for the force-measuring system; for extensometers, class 1 or better is specified for Rp0.2 and class 2 or better for properties with elongation over 5%. In Table 2, Rp0.2 denotes the 0.2% proof strength, Rm the tensile strength, A the elongation after fracture, and Z the reduction in area.

### 2.3. Microstructure Characterization

After mechanical grinding and polishing, the samples were etched with 4% nitric acid–ethanol solution for metallographic structure characterization. The microstructure of the specimens was examined using a field-emission scanning electron microscope (FEI Nova Nano SEM 450, Hillsboro, OR, USA), and elemental mapping of Cr, Ni, and C was performed using an electron probe microanalyzer (EPMA, JXA-iHP200, JEOL Ltd., Tokyo, Japan). X-ray diffraction (XRD) analysis was performed using a Rigaku (Tokyo, Japan) D-MAX 2500 diffractometer (18 kW) with Cu Kα radiation, a step size of 0.01°, and a scanning rate of 2°/min. EBSD specimens were electrolytically polished for 3 min using an electrolyte consisting of 90% acetic acid and 10% perchloric acid at a voltage of 30 V and a current of 0.5 A. EBSD characterization was performed using a ZEISS (Jena, Germany) Gemini 300 field-emission scanning electron microscope equipped with an Oxford Instruments (High Wycombe, UK) Symmetry S3 EBSD detector. Based on the grain size of the samples, a step size of 0.3 μm was selected for EBSD scanning. The acquired EBSD data were analyzed using AZtecCrystal 2.1 software. The morphology and distribution of precipitates were characterized using a transmission electron microscope (TEM, FEI Talos F200X, Hillsboro, OR, USA) equipped with a high-angle annular dark-field (HAADF) detector. TEM thin foils were prepared by mechanical thinning followed by twin-jet electropolishing, with an electrolyte composed of 10 vol% perchloric acid in ethanol at a processing temperature of −30 °C.

## 3. Results and Discussion

### 3.1. Effect of Heat Treatment Temperature on Hardness and Microstructure

**Figure 2:**
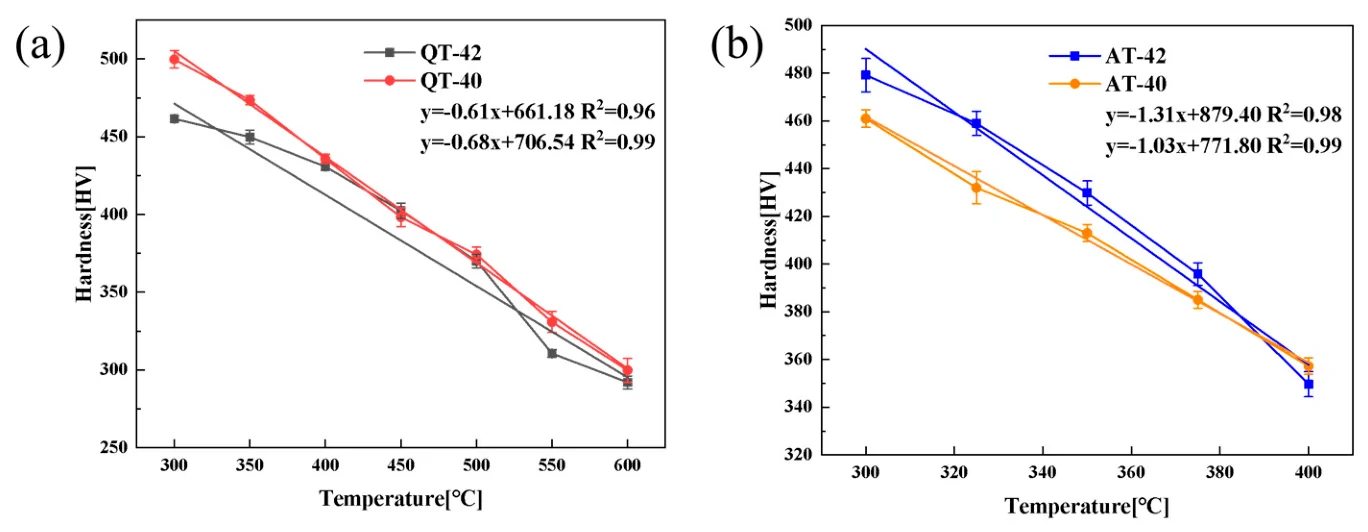
Variation in Vickers hardness [HV1] of (**a**) Q&T and (**b**) austempered 42CrMo and 40CrNiMo steels with tempering and austempering temperatures. Linear fitting equations and the corresponding coefficients of determination (R^2^) are indicated in the figure. HV1:Vickers hardness measured at a 1 kgf test force.

The microhardness measurement results of samples heat-treated at different temperatures are shown in Figure 2. The results indicate that the hardness of the samples exhibits a decreasing trend with the increase in either the Q&T or the austempering temperature. By comparison, under austempering, the hardness of 42CrMo decreases from 479 HV1 at 300 °C to 350 HV1 at 400 °C, while that of 40CrNiMo decreases from 461 HV1 to 337 HV1. Linear fitting of the hardness–temperature relationship shows that the hardness decreases nearly twice as rapidly during austempering as during Q&T. This demonstrates that the diffusion and migration rate of elements in the alloy steel under austempering conditions is higher than that under the Q&T process, which facilitates the nucleation and precipitation of carbides. This behavior may be related to the lower dislocation density and reduced lattice distortion in the austempered specimens, which provide weaker barriers to atomic diffusion [18].

**Figure 3:**
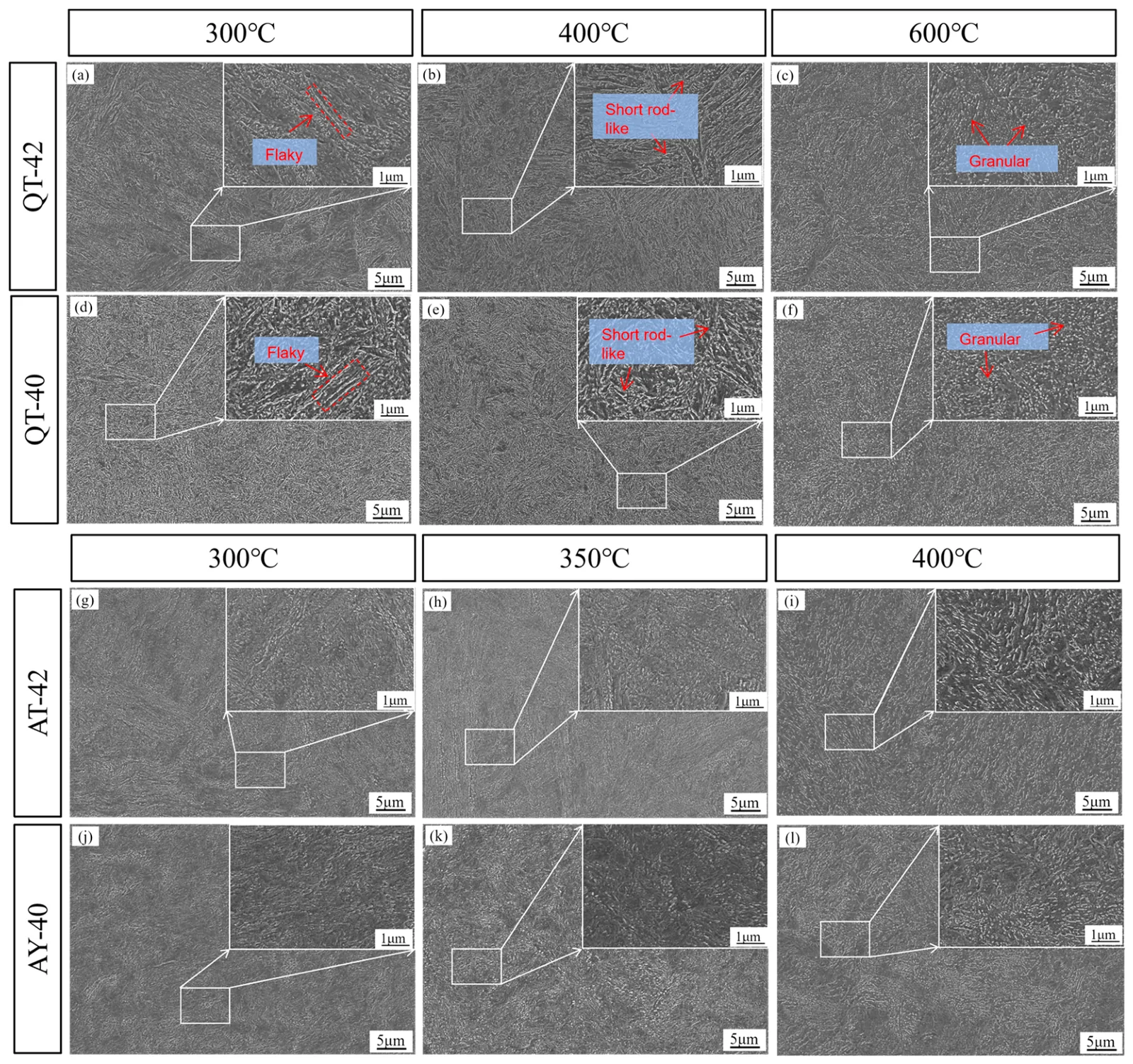
SEM micrographs of 42CrMo and 40CrNiMo steels after quenching and tempering (Q&T) or austempering (AT) at the indicated temperatures. Panels (**a**–**f**) show Q&T specimens tempered at 300, 400, and 600 °C, and panels (**g**–**l**) show AT specimens austempered at 300, 350, and 400 °C.

As shown in Figure 3, when the quenched and tempered specimens were tempered at a relatively low temperature of 300 °C, a large number of needle-like or flaky carbides precipitated along the phase interfaces of the original quenched martensite laths in both 42CrMo and 40CrNiMo specimens, resulting in a typical low-temperature tempered martensitic microstructure [19]. At a tempering temperature of 300 °C, the diffusivity of carbon is limited, the decomposition degree of martensite is relatively low, the dislocation density remains high, and thus, the material maintains a high hardness. When the tempering temperature increases to 400 °C, as shown in Figure 3b,e, the carbides primarily precipitate in the form of short rods and grains, and their size increases, forming a tempered sorbite microstructure, resulting in a decrease in material hardness. At 600 °C, the carbides in both steels became almost entirely spheroidized and underwent further coarsening, together with an increase in interparticle spacing. As a result, their effectiveness in impeding dislocation motion was reduced, and the hardness decreased to approximately 300 HV1.

After austenitizing at 860 °C followed by austempering at 300–400 °C, both steels form typical bainite structures. At 300 °C, both steels exhibit fine and densely packed bainite laths with relatively small lath-bundle sizes, while carbides are mainly present as short rods or granular precipitates [20]. Increasing the austempering temperature to 350 °C, the bainite laths gradually widen, the size of lath bundles increases, and the structure undergoes a certain degree of coarsening. When the austempering temperature further increases to 400 °C, the bainite lath structure and carbide size are significantly coarsened. With the increase in the austempering temperature, the diffusivity of carbon is enhanced, and the bainitic transformation proceeds more rapidly and extensively. The growth of carbides and the coarsening of bainite laths reduce the effects of precipitation strengthening and interface strengthening, thereby decreasing the hardness of the material.

The comparative analysis reveals that 42CrMo and 40CrNiMo steels exhibit similar hardnesses of approximately 400 HV1 after Q&T with tempering at 450 °C or austempering at 375 °C. These values fall within the hardness range cited for property class 12.9 fasteners under 380–435 HV1. This comparison is restricted to hardness only and should not be interpreted as demonstrating full compliance of the tested material or a finished bolt with property class 12.9, because the complete set of standard-required component-level properties was not evaluated in the present study. Subsequent comparisons therefore focus on these two heat-treatment conditions at comparable hardness.

### 3.2. Room-Temperature Tensile Properties and Fracture Surface Analysis

**Figure 4:**
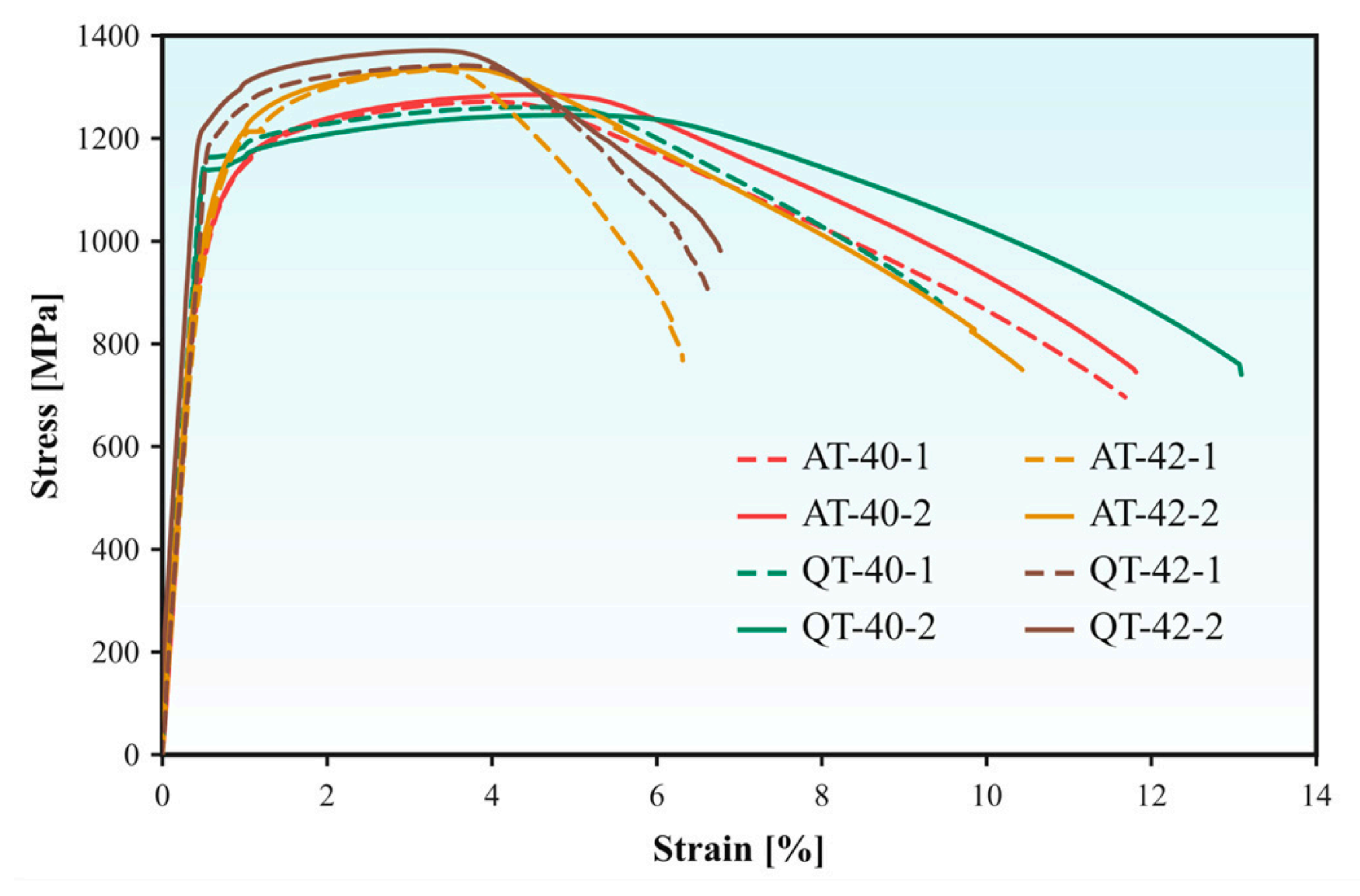
Engineering stress–strain curves and corresponding tensile properties of QT-42, AT-42, QT-40, and AT-40 specimens. Stress is expressed in MPa and strain in %.

As shown in Figure 4, after oil quenching at 860 °C and tempering at 450 °C (QT-42), 42CrMo exhibits a tensile strength of 1356 MPa, an elongation of 6.6%, and a yield strength of 1233 MPa. In contrast, after austempering treatment in a salt furnace at 375 °C (AT-42), the tensile strength remains nearly identical at 1335 MPa, but the yield strength decreases to 1100 MPa, which is approximately 133 MPa lower than that of the quenched and tempered specimens, while the elongation increases to 8.3%. Meanwhile, the reduction in area increases from 50% to 64%, indicating a significant improvement in the plasticity of the material. For 40CrNiMo specimens treated with the same quenching and tempering (QT-40) and austempering (AT-40) processes as 42CrMo, the tensile strengths are close at 1252 MPa and 1277 MPa, respectively. The yield strength also decreases from 1146 MPa in the quenched and tempered state to 1063 MPa—a reduction of about 83 MPa—while the elongation increases slightly from 11% to 11.3%. Comparative analysis reveals that under the same tensile strength condition, the austempered heat-treated specimens exhibit a lower yield strength than the quenched and tempered specimens, while the plasticity of the material is improved. Due to its higher carbon and chromium content, 42CrMo forms more carbide precipitates, resulting in approximately 60–100 MPa higher strength than 40CrNiMo. However, the addition of Ni enhances material ductility [21,22], leading to a 3% higher elongation in 40CrNiMo compared to 42CrMo.

**Figure 5:**
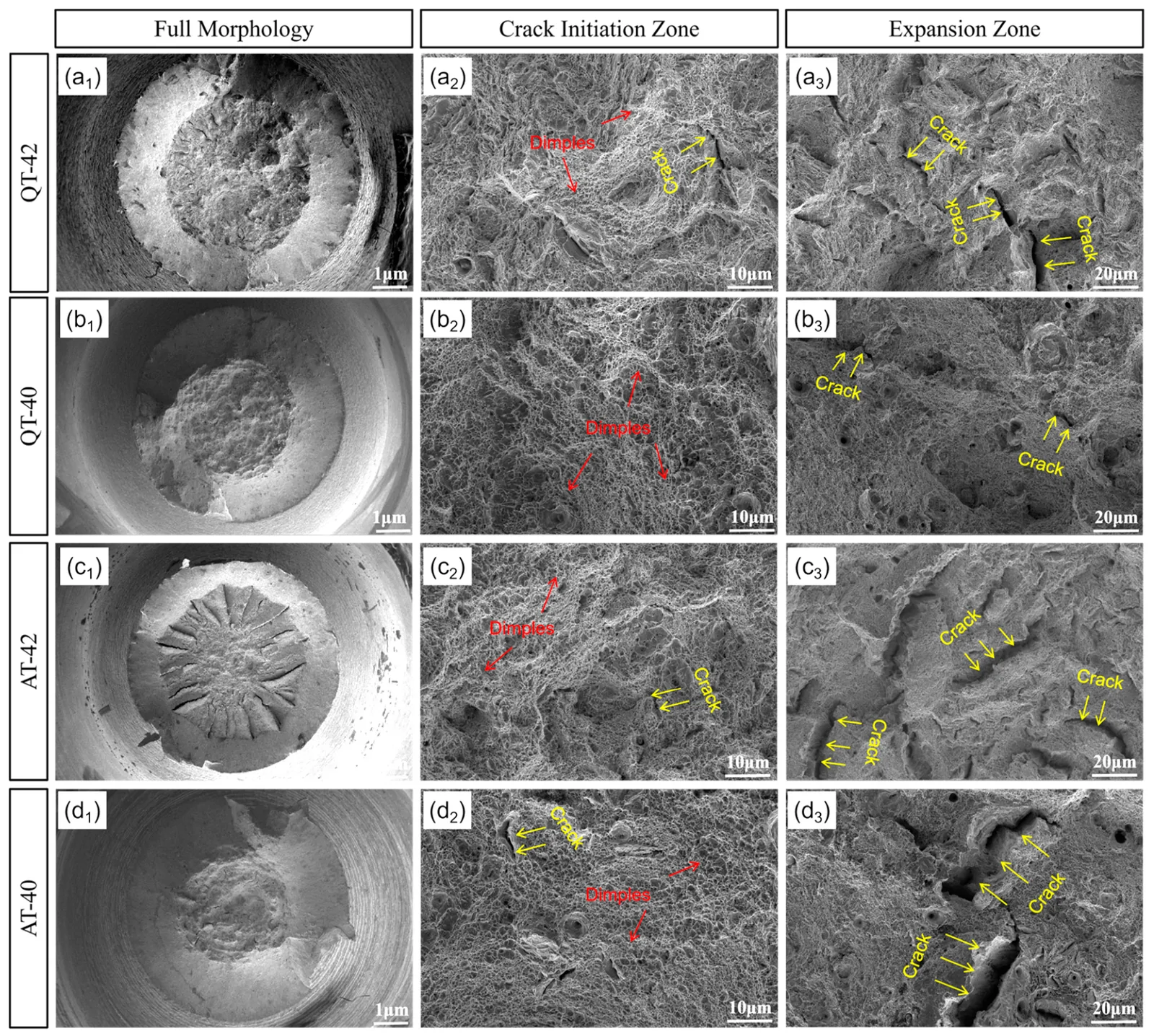
SEM micrographs of the overall tensile fracture surfaces, crack-initiation regions, and crack-propagation regions (**a_1_**–**a_3_**) QT-42 fracture surfaces; (**b_1_**–**b_3_**) AT-42 fracture surfaces; (**c_1_**–**c_3_**) QT-40 fracture surfaces; (**d_1_**–**d_3_**) AT-40 fracture surfaces.

The SEM micrographs of tensile fracture surfaces are shown in Figure 5. All specimens display typical ductile fracture characteristics, with no obvious large-scale cleavage facets or river patterns observed, indicating that fracture is primarily controlled by plastic deformation. In the crack initiation zone, numerous dimples were observed in both 42CrMo and 40CrNiMo steels. However, the dimples in 42CrMo were generally smaller and more densely distributed, which is mainly attributed to the larger number of carbides present in its microstructure, providing more nucleation sites for void formation during tensile deformation [23]. In the rapid crack expansion zone, numerous secondary cracks are observed in 42CrMo specimens, as shown in Figure 3c,d. However, the number of secondary cracks in the austempered-treated samples is fewer than in the quenched and tempered samples, reflecting improved plasticity. Moreover, the number of secondary cracks in the rapid propagation zone of 40CrNiMo is significantly lower than that in 42CrMo, as seen in Figure 5(a_3_,b_3_), indicating superior plasticity in 40CrNiMo.

### 3.3. Microstructural Characterization

**Figure 6:**
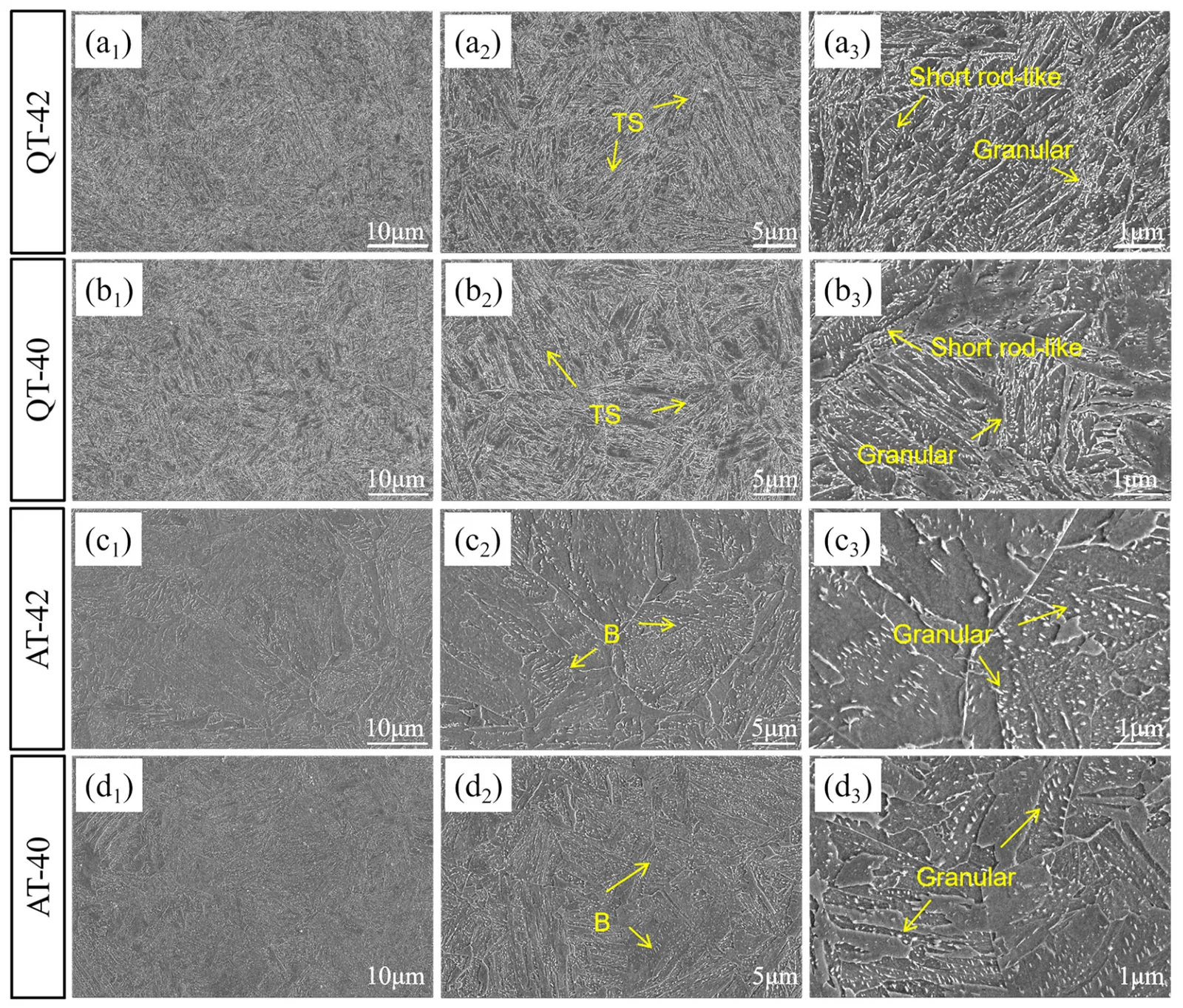
Low- and high-magnification SEM micrographs of 42CrMo and 40CrNiMo steels after Q&T at 450 °C or austempering at 375 °C, (**a_1_**–**a_3_**) QT-42; (**b_1_**–**b_3_**) QT-40; (**c_1_**–**c_3_**) AT-42; (**d_1_**–**d_3_**) AT-40. B: bainite; TS: tempered sorbite; “Granular” and “Short rod-like” refer to carbide morphologies.

The microstructures of 42CrMo and 40CrNiMo steel specimens quenched at 860 °C, tempered at 450 °C and austempered at 375 °C are shown in Figure 6. Both QT-42 and QT-40 specimens after Q&T treatment formed relatively uniform tempered sorbite structures, with carbides mainly existing in the morphology of fine granular and short rod-like. In contrast, the austempered specimens contained fewer carbide precipitates but with a comparatively larger particle size. The differences in the morphology and distribution of carbides under the two heat treatment conditions are mainly related to the microstructure transformation mechanism. After the quenching process, the microstructure of the quenched and tempered specimens is mainly martensite, with a large number of prior martensite lath interfaces and dislocations inside. These positions can provide heterogeneous nucleation sites for carbide precipitation; thus, the precipitated carbides are more dispersed and finer. During austempering at 375 °C, austenite in the specimen directly undergoes bainite transformation, and bainitic ferrite is formed via the shear mechanism [24,25], accompanied by the redistribution of carbon atoms. The lower density of carbide nucleation sites favored carbide growth, resulting in coarser carbides in the austempered specimens.

**Figure 7:**
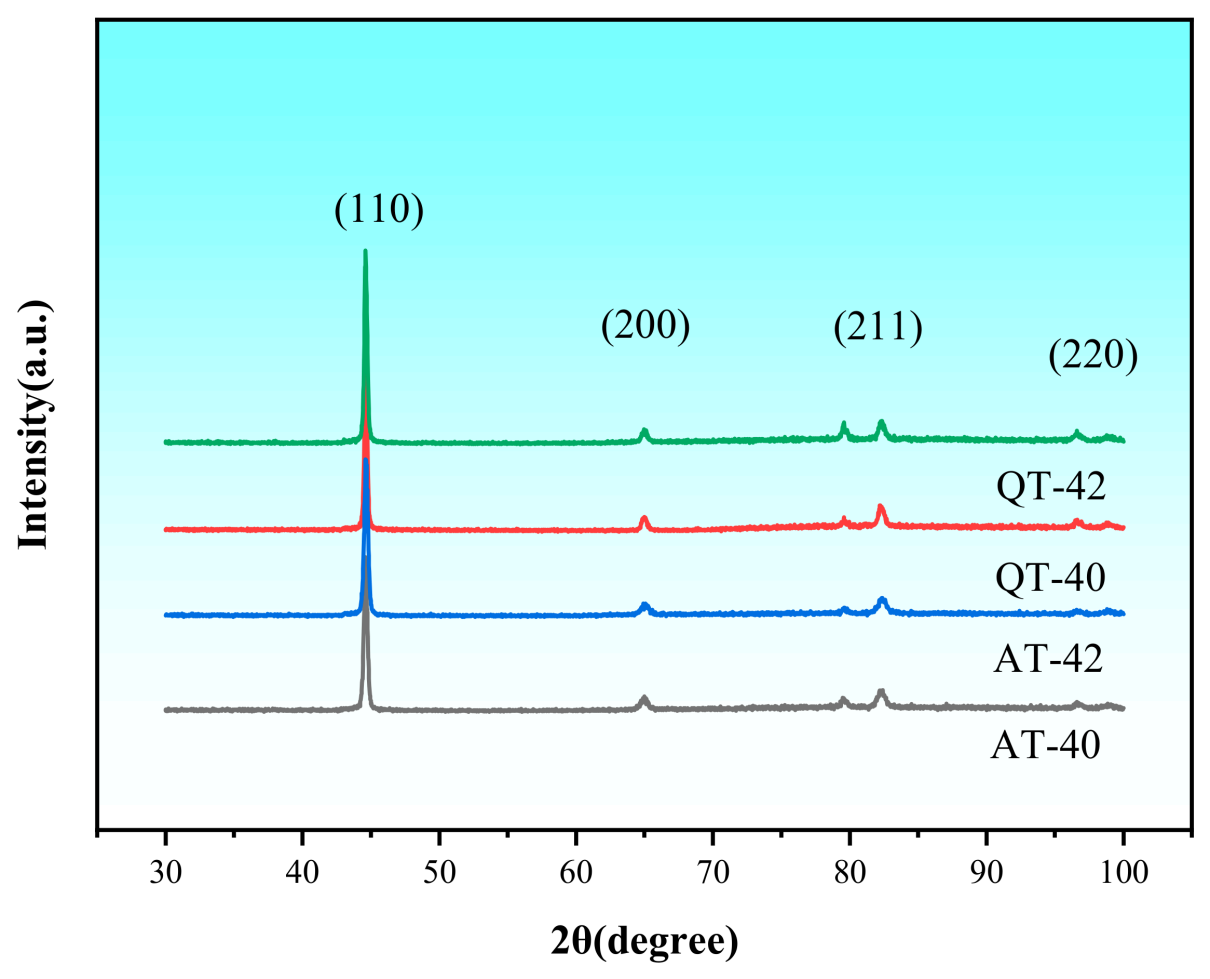
X-ray diffraction patterns of different experimental steels.

The XRD patterns of 42CrMo and 40CrNiMo specimens subjected to different heat treatment processes are shown in Figure 7. Distinct diffraction peaks appear at approximately 44.7°, 65.0°, 82.3° and 98.9° for QT-42, QT-40, AT-42 and AT-40, which correspond to the (110), (200), (211) and (220) crystallographic planes of α-Fe, respectively [26]. No diffraction peaks of retained austenite were detected. The XRD results indicate that neither austempering at 375 °C nor tempering treatment at 450 °C alters the phase composition of the material, and the matrix of the specimens is still dominated by α-Fe.

**Figure 8:**
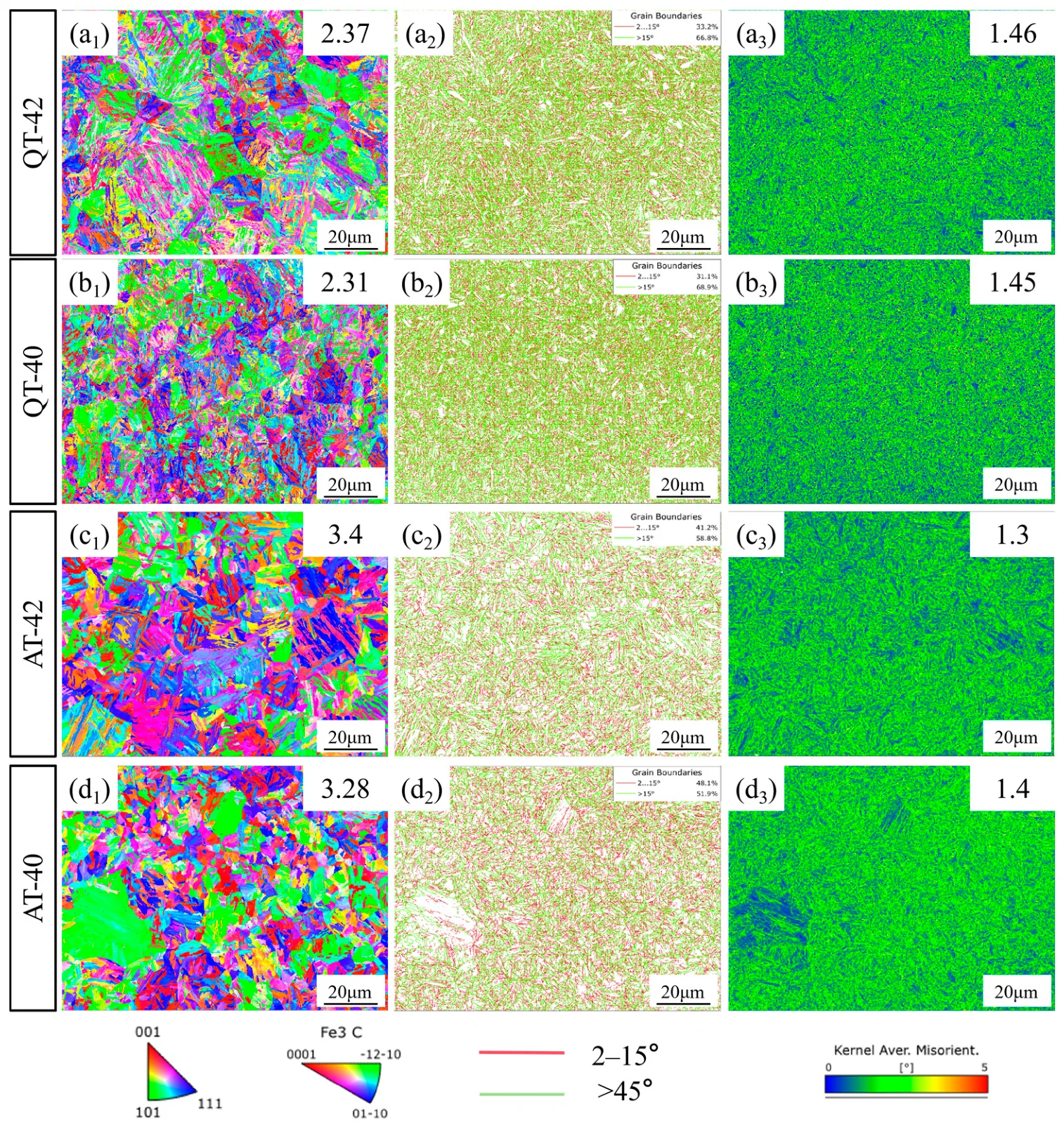
EBSD characterization of specimens subjected to different heat treatment processes: (**a_1_**–**d_1_**) IPF (inverse pole figure) orientation maps; (**a_2_**–**d_2_**) grain boundary maps delineating high-angle and low-angle grain boundaries; (**a_3_**–**d_3_**) KAM (kernel average misorientation) distribution maps.

To further reveal the influence of different heat treatment processes on the microstructure of the material, EBSD was employed to characterize the specimens, and the results are presented in Figure 8. Based on IPF orientation maps and grain size data, it can be observed that the specimens subjected to Q&T treatment and austempering treatment both retain typical lath microstructure characteristics but exhibit significant differences in grain size. The average grain sizes of the AT-42 and AT-40 specimens austempered at 375 °C are 3.28 μm and 3.40 μm, respectively, while the grain sizes of the QT-42 and QT-40 specimens tempered at 450 °C decrease to 2.31 μm and 2.37 μm, respectively. As shown in the comparison of Figure 8(a_2_–d_2_), the austempered specimens contain a higher proportion of low-angle grain boundaries (LAGBs), with the proportions of AT-42 and AT-40 being approximately 41.2% and 41.9% respectively. After Q&T treatment, the proportion of LAGBs decreases to 33.2% and 31.1%, accompanied by a significant increase in the proportion of high-angle grain boundaries. Furthermore, the KAM maps in Figure 8(a_3_–d_3_) reveal higher local misorientation in the Q&T specimens than in the austempered specimens, indicating a higher density of geometrically necessary dislocations (GNDs).

**Figure 9:**
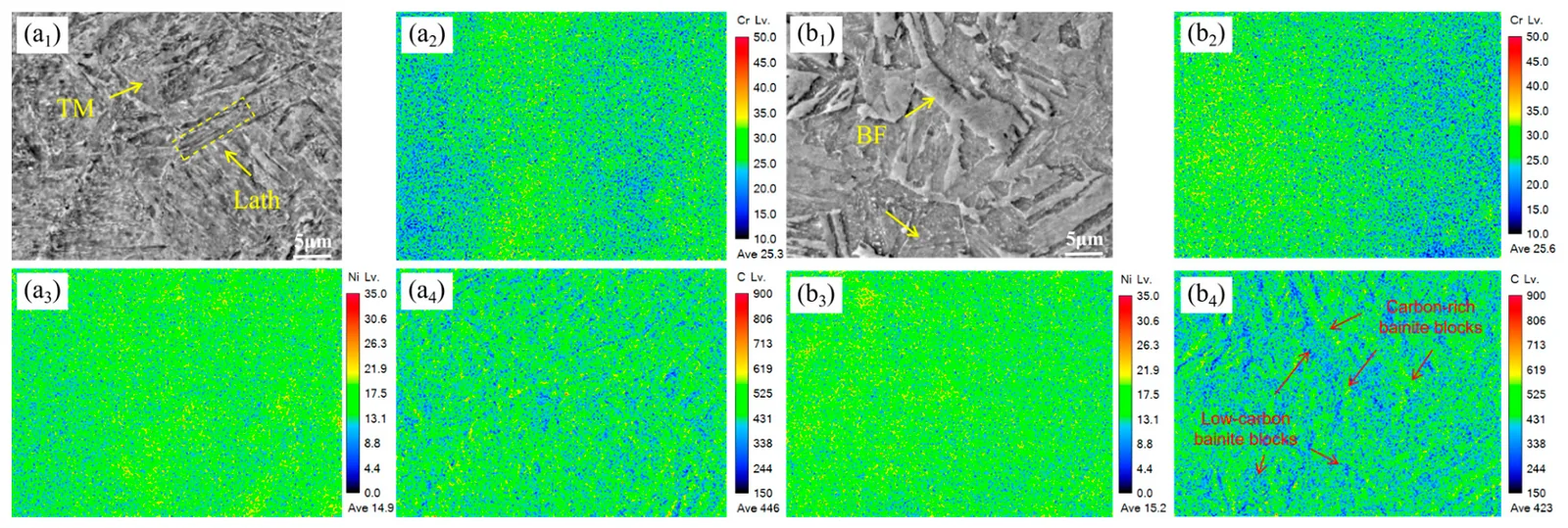
EPMA scanning images and corresponding SEM images of the C, Cr, and Ni distribution in the 40CrNiMo specimens: (**a**) quenched and tempered process; (**b**) austempered process: (**a_1_**,**b_1_**) backscattered electron microstructural images; (**a_2_**,**b_2_**) Cr distribution scanning images; (**a_3_**,**b_3_**) Ni distribution scanning images; (**a_4_**,**b_4_**) C distribution scanning images. TM: Tempered Martensite; BF: Bainitic Ferrite; Lath: Lath-shaped.

QT-40 and AT-40 specimens were selected for a comparative EPMA investigation of micro-regional elemental distributions, and the results are presented in Figure 9. As demonstrated in Figure 9(a_3_,b_3_), Ni was distributed relatively uniformly in both the austempered and the quenched and tempered structure, with no obvious enrichment regions observed. Since Ni is a non-carbide-forming element with a low diffusion rate, it is mainly dissolved in the ferrite matrix of the material in a solid solution form. However, Cr presents a certain degree of banded segregation [27,28,29], as shown in Figure 9(a_2_,b_2_). Figure 9(a_4_,b_4_) show the distribution maps of C. In the quenched and tempered specimen, the distribution of carbon is relatively uniform, and no banded segregation identical to that of Cr is detected, which indicates that the diffusion of carbon is relatively sufficient under austenitization heating at 860 °C. During quenching, carbon is dissolved in martensite in the form of solid solution and thus precipitates uniformly in the form of carbides during the subsequent tempering process. However, obvious regional differences in carbon content are observed in the austempered specimen (AT-40), which presents the characteristic of an alternating distribution of low-carbon and carbon-rich bainite blocks, as marked by the arrows in Figure 9(b_4_). This demonstrates that, during the austempering process, as the bainitic transformation proceeds, this leads to differences in the solid-solution carbon content of ferrite within bainitic colonies of different orientations. Nevertheless, the tensile mechanical property test result shows that this micro-region compositional inhomogeneity does not exert adverse effects on the strength of the material. Combined with microstructural analysis, the austempered specimens exhibited a lower number density and coarser size of carbide precipitates, particularly in the carbon-depleted regions. During tensile testing, dislocations are more prone to initiating slip, which results in a significantly lower yield strength and improved ductility compared with the quenched and tempered specimen.

**Figure 10:**
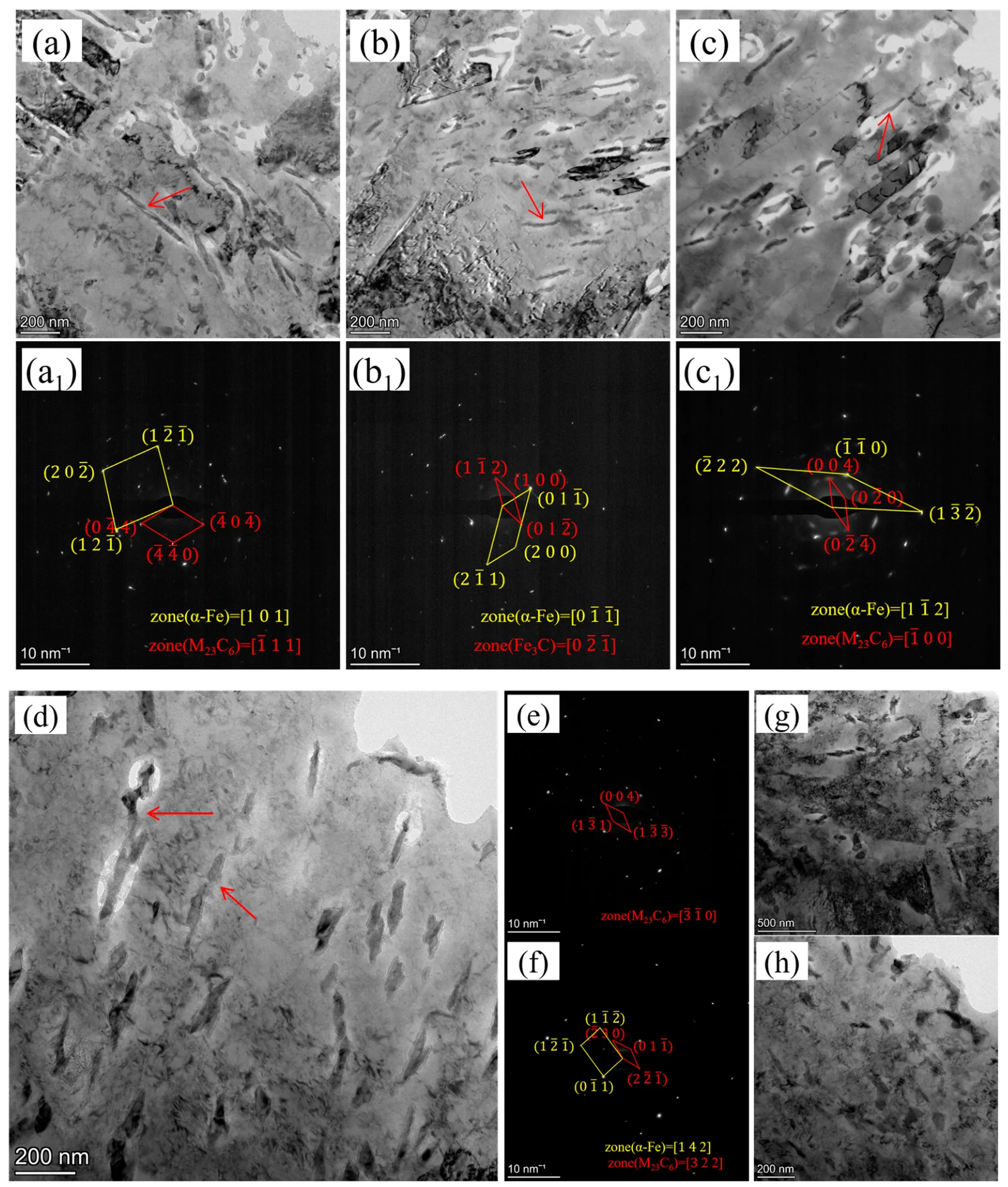
TEM characterization of carbide precipitation in the QT-40 and AT-40 specimens: (**a**–**c**) bright-field TEM images and corresponding SAED patterns (**a_1_**–**c_1_**) of QT-40; (**d**,**g**,**h**) bright-field TEM images and (**e**,**f**) SAED patterns of AT-40.

**Figure 11:**
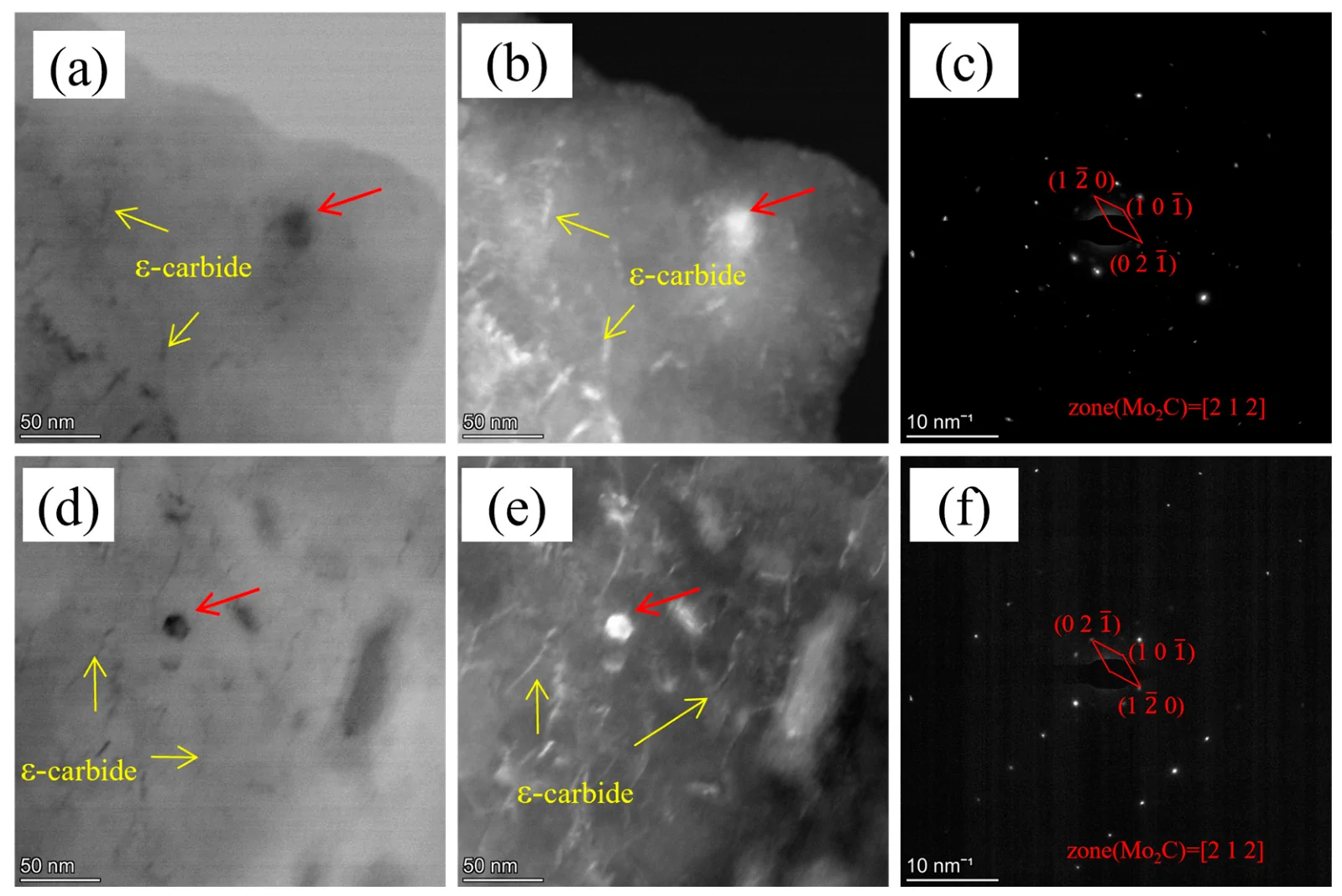
TEM characterization and SAED analysis of Mo_2_C precipitates and fine ε-carbides in the AT-40 specimen. (**a**,**d**) Bright-field images; (**b**,**e**) DF images; and (**c**,**f**) SAED patterns.

**Figure 12:**
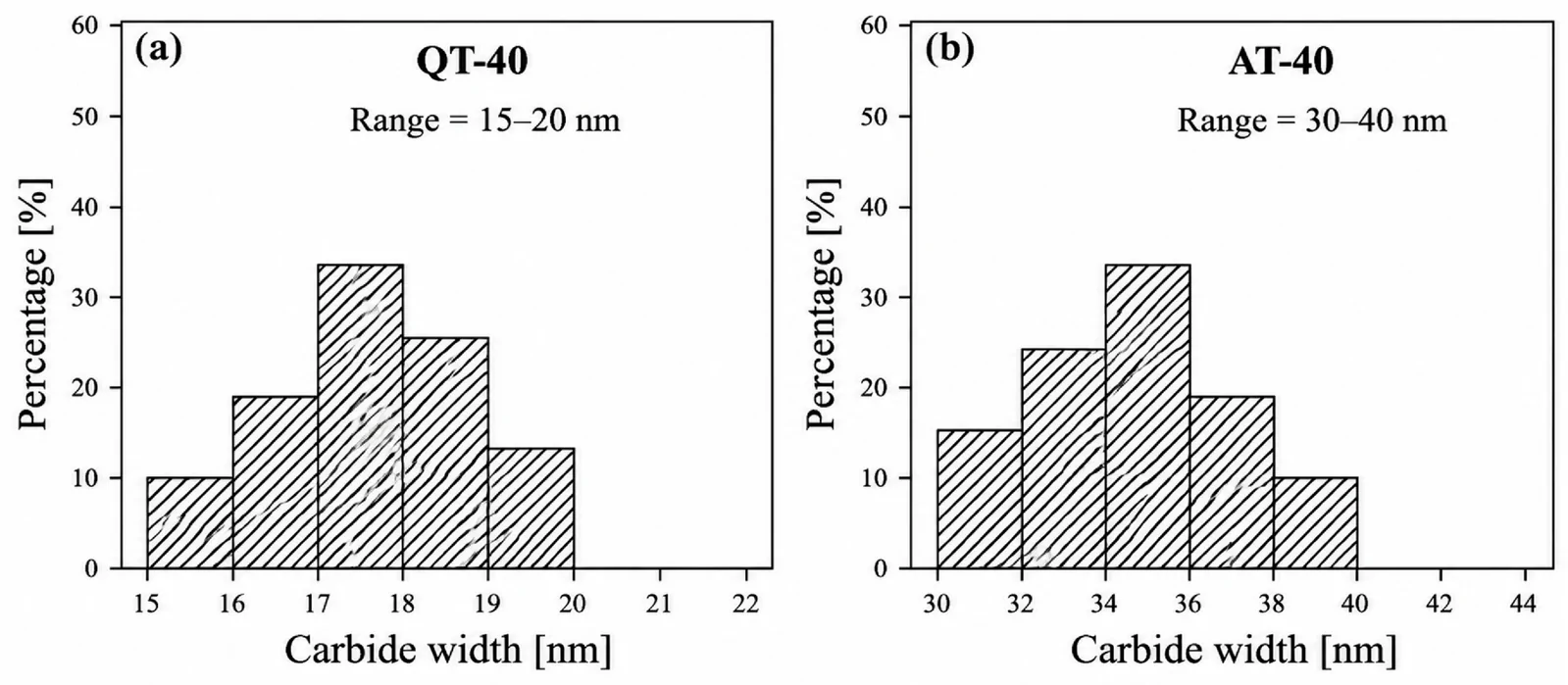
Carbide width distributions of (**a**) QT-40 and (**b**) AT-40 specimens.

TEM was employed to further characterize the carbide distribution, size and morphology of the specimens, and the results are presented in Figure 10 and Figure 11. Carbides precipitated at nanoscale in both QT-40 and AT-40 specimens. In QT-40, the carbides exhibit short rod-like or irregular granular morphologies. Since such carbides mainly nucleate and precipitate along the boundaries of prior quenched martensite, this results in a preferentially aligned and relatively uniform distribution, as shown in Figure 10a–c. In contrast, for the AT-40 specimen, apart from the majority of carbides that distribute parallel to the lath interfaces of bainitic ferrite (Figure 10d), the carbides in some bainite blocks are more nearly spherical and are randomly distributed within the matrix. The width of rod-shaped carbides or the diameter of spherical carbides in the AT-40 specimen ranges from 30 nm to 40 nm, which is larger than that of the carbides in the QT-40 specimen (15 nm to 20 nm), as shown in Figure 12. SAED (selected-area electron diffraction) pattern indexing results indicate that the dominant carbides in both QT-40 and AT-40 are face-centered cubic M_23_C_6_ type carbides [30].

As shown in Figure 11, in addition to a large amount of M_23_C_6_-type carbides, spherical carbides with sizes of 20–30 nm were also observed precipitating in QT-40 and AT-40 specimens. SAED analysis results (Figure 11c,f) confirm that these precipitates are Mo_2_C with a hexagonal structure. Furthermore, extremely fine acicular ε-carbides were detected in the bainitic ferrite laths of the austempered AT-40 specimen, as shown in Figure 11b,e.

Overall, the TEM results reveal distinct carbide precipitation characteristics between the two heat treatment conditions: Q&T promotes fine carbides preferentially distributed along prior martensite lath boundaries, whereas austempering produces relatively coarser carbides with a less directional distribution, together with a small amount of ε-carbides within the bainitic ferrite laths.

### 3.4. Impact Fracture Surface Analysis

**Figure 13:**
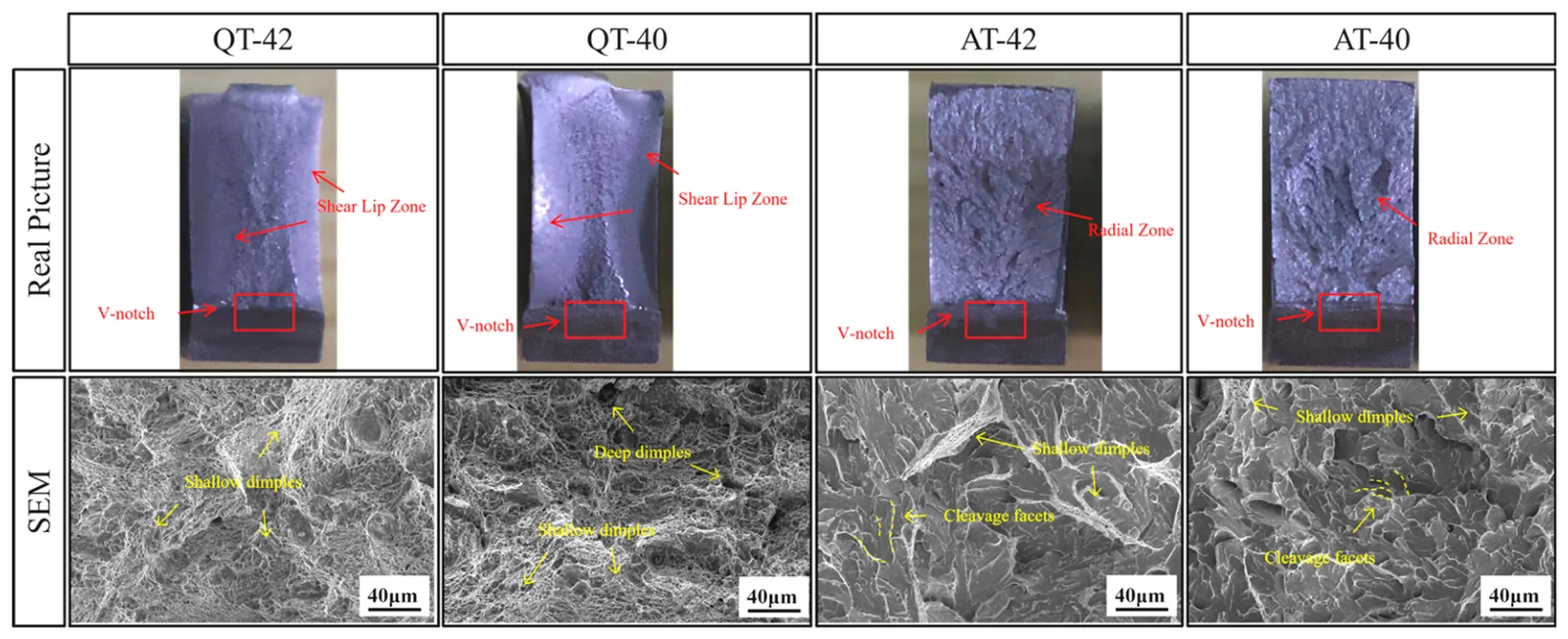
Macroscopic photographs and SEM fractographs of Charpy impact-fracture surfaces from QT-42, QT-40, AT-42, and AT-40 specimens. The images show the overall fracture morphology and higher-magnification fracture-surface features.

A pronounced reduction in impact toughness is observed after austempering. The impact energy decreases from 50.0 and 42.2 J for QT-42 to 16.0 and 16.2 J for AT-42, and from 68.2 and 74.4 J for QT-40 to 31.2 and 25.2 J for AT-40, corresponding to reductions of approximately 65% and 60%, respectively. Notably, this decrease occurs despite the comparable hardness and tensile strength of the Q&T-treated and austempered specimens. The macroscopic fracture morphologies and high-magnification SEM analysis results are shown in Figure 13. The analysis results of fracture microstructure show that a large number of dimples are distributed on the fracture surfaces of quenched and tempered samples QT-42 and QT-40. These dimples can absorb more impact energy through the processes of microvoid nucleation, growth and coalescence, and the samples also possess more pronounced shear lips, indicating that the materials have excellent toughness [31,32,33]. In contrast, the impact fracture surfaces of the austempered samples AT-42 and AT-40 are dominated by radial zones, and the fracture surfaces are mainly composed of quasi-cleavage river patterns interspersed with fine dimples, showing obvious quasi-cleavage fracture characteristics.

**Figure 14:**
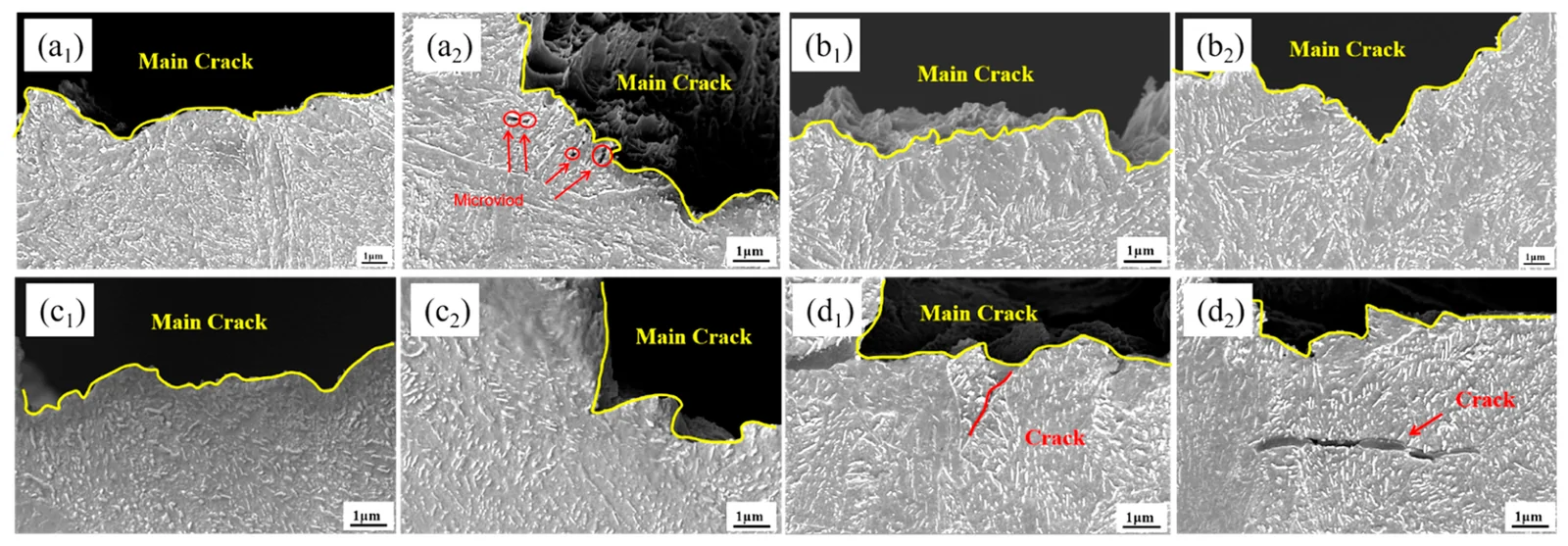
SEM images of the longitudinal sections of impact-fracture specimens: (**a_1_**,**a_2_**) QT-42; (**b_1_**,**b_2_**) QT-40; (**c_1_**,**c_2_**) AT-42; (**d_1_**,**d_2_**) AT-40.

The comparison of the microstructures on the longitudinal sections of impact fracture surfaces is shown in Figure 14. Comparative analysis reveals differences in the propagation paths of the main cracks among specimens subjected to different heat treatments. For the quenched and tempered specimens QT-42 and QT-40, the main crack outlines are relatively zigzag, and the crack propagation paths deflect multiple times, which requires higher energy consumption. Crack blunting is observed locally, no obvious secondary cracks are detected, and microvoids formed at fine carbides are observed on the fracture surface, as shown in panel Figure 14(a_2_). In contrast, for the austempered specimens AT-42 and AT-40, the main cracks propagate along relatively straight paths with little change in crack propagation direction, indicating that the cracks encounter limited resistance during extension [34,35,36,37,38,39], and secondary cracks extending along bainite block boundaries can be observed locally (Figure 14(d_1_)). Compared with the quenched and tempered specimens, the austempered specimens AT-42 and AT-40 have coarser grains and larger carbide particles. Meanwhile, microscale carbon segregation leads to different hardnesses among bainite blocks, which facilitates the initiation and rapid extension of impact cracks at bainite blocks or lath interfaces, consequently reducing the impact toughness of the material. This difference in crack propagation behavior also explains why the improved tensile ductility of the austempered specimens does not lead to higher absorbed impact energy. Under tensile loading, the austempered specimens can undergo higher plastic deformation, whereas under impact loading, the relatively straight crack paths and secondary cracking along bainite block boundaries facilitate rapid crack propagation. Consequently, improved tensile ductility and reduced absorbed impact energy can coexist in the austempered specimens.

**Figure 15:**
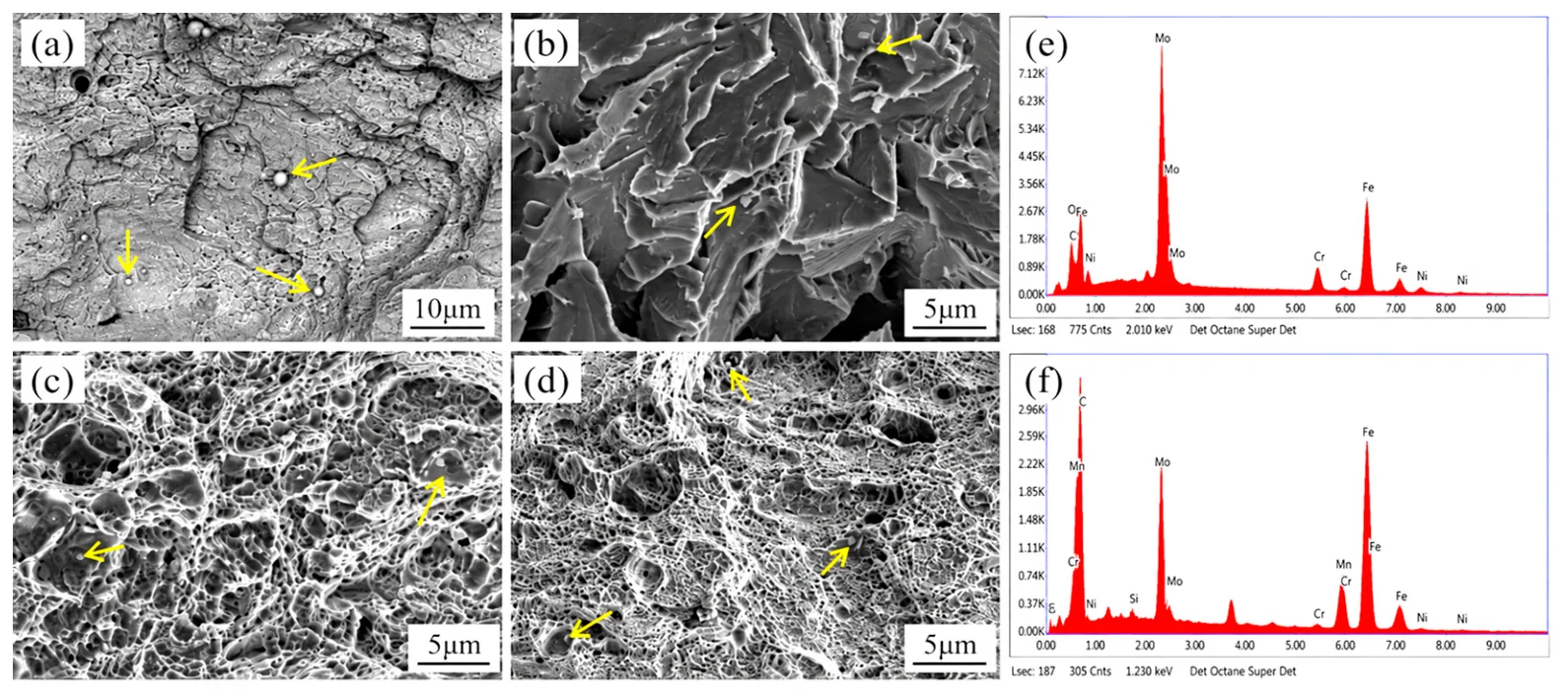
SEM–EDS analysis of precipitates on impact-fracture surfaces: (**a**) backscattered-electron SEM image of AT-40; (**b**) AT-42; (**c**) QT-40; (**d**) QT-42; (**e**,**f**) EDS spectra from Mo-enriched particles.

As shown in Figure 15, a small number of spherical precipitates with a large size of 0.2–1 μm were detected on the impact fracture surface, and the precipitates in the austempered samples exhibited larger dimensions in particular. Results from SEM coupled with EDS reveal that these granules are enriched with Mo and are primarily Mo-containing carbides. During the austempering process, the bainite transformation is accompanied by continuous redistribution of carbon atoms, which leads to the formation of regions with high local carbon concentration and provides the driving force for the nucleation of Mo carbides. Meanwhile, the relatively low defect density in bainitic microstructure reduces the number of carbide nucleation sites, promoting the migration of Mo atoms to existing granules and facilitating their growth. This process results in the coarsening and eventual spheroidization of Mo-enriched granules [40,41,42]. Under impact loading, these coarse particles can readily induce stress concentration at the particle matrix interfaces, thereby promoting crack initiation and reducing impact toughness.

**Figure 16:**
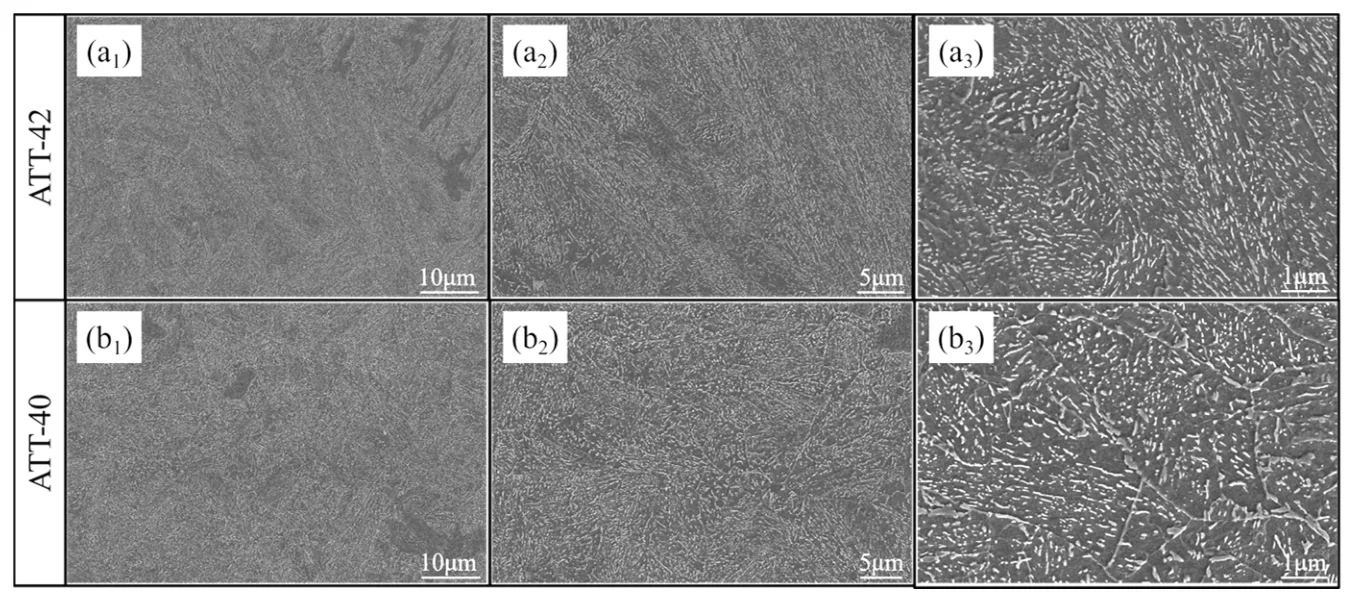
SEM micrographs of 42CrMo and 40CrNiMo specimens after austempering at 375 °C followed by tempering at 450 °C: (**a_1_**–**a_3_**) ATT-42 and (**b_1_**–**b_3_**) ATT-40.

The austempered specimens were further subjected to tempering treatment at 450 °C with a holding time of 1 h. The impact toughness of the 40CrNiMo specimen (ATT-40) increased significantly from 25.2 J to 67 J, while that of the 42CrMo specimen (ATT-42) increased by 14 J. Rockwell hardness tests revealed that the hardness of both ATT-42 and ATT-40 specimens decreased notably, with reductions of 2 HRC (Rockwell C-scale hardness) and 5 HRC, respectively. SEM observations of the microstructure (as shown in Figure 16) show that the carbide precipitation in ATT-42 and ATT-40 specimens is more sufficient than that in the austempered AT-42 and AT-40 specimens. Meanwhile, the solute carbon in bainitic ferrite precipitates on the basis of the carbides formed in the original austempering process, which further promotes the coarsening of carbide size, thus leading to the decrease in the hardness of the specimens.

## 4. Conclusions

(1) After austenitizing at 860 °C followed by oil quenching and tempering at temperatures ranging from 300 °C to 600 °C, 42CrMo and 40CrNiMo steels primarily form tempered martensite or tempered sorbite microstructure. In contrast, austempering performed at 300–400 °C yields typical bainitic lath microstructure. Increasing the treatment temperature decreases the hardness of both steels, with a more pronounced reduction under austempering. After Q&T treatment with tempering at 450 °C or austempering at 375 °C, both steels exhibit comparable hardness satisfying the requirements for grade 12.9 high-strength bolts.
(2) Q&T produces finer and more uniformly distributed carbides, whereas austempering results in fewer and coarser carbides associated with the bainitic structure. The carbide structure in both types of specimens is predominantly of the M_23_C_6_ type. Extremely fine ε-carbides are additionally observed within the bainite ferrite laths. Furthermore, austempering results in relatively coarser grains and heterogeneous carbon distribution among bainite blocks. These microstructural characteristics provide the basis for the different mechanical responses of the two heat-treatment conditions.
(3) At comparable tensile strength, austempering reduces the yield strength and improves the ductility of both steels, which is associated with the lower amount and coarser size of carbide precipitates. However, austempering also leads to a substantial decrease in absorbed impact energy, which is mainly attributed to the fact that cracks are more likely to initiate and propagate along bainite blocks or lath interfaces. Subsequent tempering improves the impact performance but is accompanied by a reduction in hardness.

From an engineering perspective, austempering provides a potential route for tailoring the strength–ductility balance of high-strength fastener steels; however, its reduced absorbed impact energy should be considered for impact-loaded applications. Future work should focus on optimizing the austempering parameters to improve the balance among strength, ductility, and impact performance, with particular attention to controlling carbide precipitation and microstructural heterogeneity and their effects on crack propagation.

## Figures and Tables

**Table 1:** Chemical compositions of 42CrMo and 40CrNiMo steels [wt.%].

	C	Cr	Ni	Si	Mn	Mo
42CrMo	0.42	1.02	≤0.30	0.25	0.74	0.22
40CrNiMo	0.40	0.85	1.35	0.31	0.69	0.20

**Table 2:** Tensile test data.

Sample	Rp0.2 [MPa]	Rm [MPa]	A [%]	Z [%]
QT-42-1	1224	1342	6.6	53
QT-42-2	1242	1371	6.7	47
Mean	1233	1356	6.6	50
QT-40-1	1152	1260	9.4	61
QT-40-2	1140	1245	12.78	60
Mean	1146	1252	11.09	60.5
AT-42-1	1099	1333	6.31	66
AT-42-2	1102	1338	10.13	62
Mean	1100	1335	8.22	64
AT-40-1	1077	1271	11.4	61
AT-40-2	1049	1284	11.5	61
Mean	1063	1277	11.45	61

## Data Availability

The original contributions presented in this study are included in the article. Further inquiries can be directed to the corresponding author.
